# Supplementary material for: COZOID: contact zone identifier for visual analysis of protein-protein interactions
Source: BMC Bioinformatics. 2018 Apr 6;19:125. doi: 10.1186/s12859-018-2113-6 (PMC5889581; doi:10.1186/s12859-018-2113-6)
Supplement: Supplementary file 2 — Software build. Executable binary file of the software tool. (ZIP 118,784 kb) [file 12859_2018_2113_MOESM2_ESM.zip › cozoid/java/docs/beansbinding-1.2.1-doc.zip!/org/jdesktop/beansbinding/AbstractBindingListener.html]

AbstractBindingListener (Beans Binding)


---


|  |  |  |
| --- | --- | --- |
| **Overview** \| **Package** \| **Class** \| **Use** \| **Tree** \| **Deprecated** \| **Index** \| **Help** | |  |
| PREV CLASS   **NEXT CLASS** | **FRAMES**    **NO FRAMES**     **All Classes** |  |
| SUMMARY: NESTED \| FIELD \| CONSTR \| METHOD | DETAIL: FIELD \| CONSTR \| METHOD |  |


---


## org.jdesktop.beansbinding Class AbstractBindingListener

```
java.lang.Object
  org.jdesktop.beansbinding.AbstractBindingListener
```

**All Implemented Interfaces:**: java.util.EventListener, BindingListener

---

``` public abstract class AbstractBindingListener extends java.lang.Object implements BindingListener ```

An abstract subclass of `BindingListener` that simplifies writing
`BindingListeners` by allowing you to extend this class and re-implement
only the methods you care about.

---

**Constructor Summary**

`AbstractBindingListener()`


| **Method Summary** | |
| --- | --- |
| `void` | `bindingBecameBound(Binding binding)`             Notification that a `Binding` has been bound. |
| `void` | `bindingBecameUnbound(Binding binding)`             Notification that a `Binding` has been unbound. |
| `void` | `sourceChanged(Binding binding, PropertyStateEvent event)`             Notification that the source property of a `Binding` has fired a `PropertyStateEvent` for the `Binding's` source object. |
| `void` | `sourceEdited(Binding binding)`             **Deprecated.** *This method has been replaced by `sourceChanged(org.jdesktop.beansbinding.Binding, org.jdesktop.beansbinding.PropertyStateEvent)` and it will go away soon. It is being kept for a short period only, to assist in migration.* |
| `void` | `synced(Binding binding)`             Notification that the source and target of a `Binding` have been made in sync. |
| `void` | `syncFailed(Binding binding, Binding.SyncFailure failure)`             Notification that the `Binding` attempted to sync the source and target, but the sync failed. |
| `void` | `targetChanged(Binding binding, PropertyStateEvent event)`             Notification that the target property of a `Binding` has fired a `PropertyStateEvent` for the `Binding's` target object. |
| `void` | `targetEdited(Binding binding)`             **Deprecated.** *This method has been replaced by `targetChanged(org.jdesktop.beansbinding.Binding, org.jdesktop.beansbinding.PropertyStateEvent)` and it will go away soon. It is being kept for a short period only, to assist in migration.* |

**Methods inherited from class java.lang.Object**

`clone, equals, finalize, getClass, hashCode, notify, notifyAll, toString, wait, wait, wait`

**Constructor Detail**

### AbstractBindingListener

```
public AbstractBindingListener()
```


**Method Detail**

### bindingBecameBound

```
public void bindingBecameBound(Binding binding)
```

:   Notification that a `Binding` has been bound.

    :   **Specified by:**: `bindingBecameBound` in interface `BindingListener`
    :   **Parameters:**: `binding` - the `Binding`

---


### bindingBecameUnbound

```
public void bindingBecameUnbound(Binding binding)
```

:   Notification that a `Binding` has been unbound.

    :   **Specified by:**: `bindingBecameUnbound` in interface `BindingListener`
    :   **Parameters:**: `binding` - the `Binding`

---


### syncFailed

```
public void syncFailed(Binding binding,
                       Binding.SyncFailure failure)
```

:   Notification that the `Binding` attempted to sync the source and
    target, but the sync failed.

    :   **Specified by:**: `syncFailed` in interface `BindingListener`
    :   **Parameters:**: `binding` - the `Binding`: `failure` - the reason the sync failed

---


### synced

```
public void synced(Binding binding)
```

:   Notification that the source and target of a `Binding` have
    been made in sync.

    :   **Specified by:**: `synced` in interface `BindingListener`
    :   **Parameters:**: `binding` - the `Binding`

---


### sourceChanged

```
public void sourceChanged(Binding binding,
                          PropertyStateEvent event)
```

:   Notification that the source property of a `Binding` has fired
    a `PropertyStateEvent` for the `Binding's` source object.

    This implementation calls `sourceEdited` if the provided event returns
    `true` from `getValueChanged`.

    :   **Specified by:**: `sourceChanged` in interface `BindingListener`
    :   **Parameters:**: `binding` - the `Binding`

---


### targetChanged

```
public void targetChanged(Binding binding,
                          PropertyStateEvent event)
```

:   Notification that the target property of a `Binding` has fired
    a `PropertyStateEvent` for the `Binding's` target object.

    This implementation calls `targetEdited` if the provided event returns
    `true` from `getValueChanged`.

    :   **Specified by:**: `targetChanged` in interface `BindingListener`
    :   **Parameters:**: `binding` - the `Binding`

---


### sourceEdited

```
@Deprecated
public void sourceEdited(Binding binding)
```

:   **Deprecated.** *This method has been replaced by `sourceChanged(org.jdesktop.beansbinding.Binding, org.jdesktop.beansbinding.PropertyStateEvent)` and it
    will go away soon. It is being kept for a short period only,
    to assist in migration.*

    :   Notification that the source property of a `Binding` has fired
        a `PropertyStateEvent` indicating that its **value or readability**
        has changed for the `Binding's` source object. Called by the default
        `AbstractBindingListener's` implementation of `sourceChanged`.

        :   **Parameters:**: `binding` - the `Binding`

---


### targetEdited

```
@Deprecated
public void targetEdited(Binding binding)
```

:   **Deprecated.** *This method has been replaced by `targetChanged(org.jdesktop.beansbinding.Binding, org.jdesktop.beansbinding.PropertyStateEvent)` and it
    will go away soon. It is being kept for a short period only,
    to assist in migration.*

    :   Notification that the target property of a `Binding` has fired
        a `PropertyStateEvent` indicating that its **value or readability**
        has changed for the `Binding's` target object. Called by the default
        `AbstractBindingListener's` implementation of `targetChanged`.

        :   **Parameters:**: `binding` - the `Binding`


---


|  |  |  |
| --- | --- | --- |
| **Overview** \| **Package** \| **Class** \| **Use** \| **Tree** \| **Deprecated** \| **Index** \| **Help** | |  |
| PREV CLASS   **NEXT CLASS** | **FRAMES**    **NO FRAMES**     **All Classes** |  |
| SUMMARY: NESTED \| FIELD \| CONSTR \| METHOD | DETAIL: FIELD \| CONSTR \| METHOD |  |


---
